# Supplementary material for: WordCommentsAnalyzer: A windows software tool for qualitative research
Source: F1000Res. 2018 Sep 4;7:536. Originally published 2018 May 3. [Version 2] doi: 10.12688/f1000research.14819.2 (PMC6137411; doi:10.12688/f1000research.14819.2)
Supplement: Supplementary file 3 [file f1000research-7-17614-s0002.tgz › b2a79496-4ada-47c4-9682-a99ce867836d.zip!/Eight Iranian Health Journals/Salmand_Iranian Journal of Ageing _20.docx]

© Salmand_Iranian Journal of Ageing . This work is licensed under CC BY-NC 4.0 (https://creativecommons.org/licenses/by-nc/4.0/).

Volume & Issue: دوره 8 - شماره 2

Title: اثربخشی گشتالت درمانی بر شادکامی سالمندان

http://salmandj.uswr.ac.ir/article-1-650-fa.html

اهداف: این مطالعه با هدف تعیین اثربخشی گشتالت درمانی بر افزایش شادکامی سالمندان انجام شده است. مواد و روش‌ها: طرح پژوهشی حاضر از نوع آزمایشی با پیش‌آزمون-پس‌آزمون و گروه کنترل است. جامعه آماری شامل سالمندان عضو کانون جهان‌دیدگان شاد بود. با استفاده از چک لیست ملاک‌های ورود و خروج و اجرای آزمون کوتاه شناختی تعداد 50 نفر از سالمندان مورد ارزیابی و از میان آنان 28 نفر واجد شرایط ورود به مطالعه انتخاب شده و به روش تصادفی در دو گروه آزمایش و کنترل قرار گرفتند. مقیاس شادکامی آکسفورد برای دو گروه اجرا شد. جلسات گشتالت درمانی برای گروه مداخله به‌صورت هفتگی در هشت جلسه برگزار گردید. پس از جمع آوری داده‌های پس آزمون، تجزیه و تحلیل داده‌ها با استفاده از آزمون آماری t برای گروه‌های مستقل انجام شد. یافته‌ها: نتایج نشان داد که تفاوت میانگین نمرات شادکامی دو گروه معنی‌دار است و گشتالت درمانی به‌صورت معنی‌دار موجب افزایش شادکامی سالمندان می‌شود (P<0/01). همچنین مقایسه نمرات میانگین پیش‌آزمون و پس‌آزمون در دو گروه حاکی از افزایش نمرات شادکامی در کلیه خرده مقیاس‌ها در مقطع پس‌آزمون با اختلاف معنی‌دار بود (P=0/000). نتیجه‌گیری: نتایج این پژوهش نشان داد که گشتالت درمانی می‌تواند در ارتقای عواطف مثبت و شادکامی سالمندان مفید باشد. اجرای چنین جلسات گروهی در اقامتگاه‌ها و مراکز تجمع سالمندان توصیه می‌شود.

Title: بررسی تاثیر برنامه‌های اجتماع‌محور بر رفاه ذهنی سالمندان

http://salmandj.uswr.ac.ir/article-1-562-fa.html

اهداف: افزایش پیشرونده جمعیت سالمند در بسیاری از کشورهای توسعه یافته موجب معطوف شدن توجه به مسایل این گروه سنی شده است. از جمله روشهایی که در دهه‌های اخیر برای مقابله با مشکلات سالمندان و دیگر گروه‌های خاص به کار گرفته شده، برنامه‌های اجتماع‌محور است. از آنجا که اجرای چنین برنامه‌هایی مراحل اولیه رشد خود را در کشورهای مختلف می‌گذراند، لذا هدف از پژوهش بررسی برنامه‌های اجتماع‌محور در میان سالمندان کهریزک و تاثیر آن بر میزان رفاه ذهنی آنان است. مواد و روش‌ها: در این پژوهش 100 نفر از سالمندان 60 سال به بالای آسایشگاه کهریزک (شعبه کرج) به روش نمونه‌گیری کاملا تصادفی انتخاب شدند. این افراد در دوگروه 50 نفری آزمون و شاهد قرار گرفتند. ویژگی اصلی تفکیک دو گروه، شرکت در برنامه‌های اجتماع‌محور بوده است. روش بررسی به صورت مداخله‌ای بوده که با استفاده از تکنیک پرسش‌نامه و مصاحبه اطلاعات مورد نیاز جمع‌آوری و برای تحلیل داده‌ها از نرم‌افزار SPSS استفاده شده است. یافته‌ها: نتایج نشان‌دهنده تفاوت معنادار میان دو گروه آزمون و شاهد است. میانگین رفاه ذهنی گروه آزمون 2/55 و میانگین گروه شاهد 1/34- بوده است. سالمندانی که در برنامه‌های اجتماع‌محور شرکت داشته و خدمات معناداری را دریافت کرده‌اند، در بررسی شاخص‌های رفاه ذهنی میزان بالاتری از شادکامی، رضایت از زندگی و کیفیت زندگی را داشته‌اند. نتیجه‌گیری: با وجودی که مدت زمان زیادی از اجرای برنامه‌های اجتماع‌محور در کهریزک نمی‌گذرد، اما همان‌طوری‌که در نظریه‌ها و مدل‌های نظری مشخص شده افراد شرکت‌کننده در این برنامه‌ها با بازگشت به اجتماع و انجام فعالیت‌های مختلف هویت فردی و اجتماعی خویش را باز یافته و توانسته‌اند در مقایسه با دیگر سالمندان از میزان رفاه ذهنی بالاتری در ابعاد احساس شادکامی، کیفیت زندگی و رضایت از زندگی برخوردار شوند.

Title: تعیین اپیدمیولوژی سقوط در سالمندان ساکن مراکز نگهداری شهر تهران - 1390

http://salmandj.uswr.ac.ir/article-1-521-fa.html

اهداف: سقوط یکی از شایع ترین مصدومیت‌های تکرارشونده درسالمندان، به‌ویژه سالمندان مقیم در مراکز نگهداری است. این مطالعه با هدف تعیین اپیدمیولوژی سقوط در زنان و مردان سالمند ساکن در مراکز نگهداری شهر تهران در سال 1390 انجام شده است. مواد و روش‌ها: در یک مطالعه مقطعی از بین کلیه سالمندان مقیم مراکز نگهداری شهر تهران تعداد 400 سالمند 60 سال و بالاتر به صورت تصادفی ساده انتخاب شدند. اطلاعات با استفاده از مشاهده، مصاحبه، مطالعه پرونده و پرسش‌نامه‌های استاندارد: فعالیت‌های روزانه زندگی با ایندکسبارتل(Activities Daily Living Barthel Index)، تعادل و راه رفتن بامقیاس تعادل برگ (Berg Balance Scale)، ترس از سقوط با مقیاس(FES)Falls efficacy scale، وضعیت تغذیه با پرسش‌نامه (MNA)MiniNutritional Assessment توسط کارشناسان با آموزش یکسان پس از کسب رضایت از نمونه‌ها جمع‌آوری شد. عوامل محیطی با مشاهده فضای داخلی و خارجی مرکز نگهداری و استفاده از چک لیست ثبت شد. یافته‌ها: از تعداد کل 400 سالمند مورد بررسی، %35  زن و %65 مرد با میانگین و انحراف معیار سنی 22.0±70 سال بودند. 73درصد سابقه سقوط طی یکسال گذشته داشتند و 60درصد بیش از یک بار سقوط را گزارش کرده‌اند و 41درصد از آن‌ها در گروه سنی 89-80 بودند (71درصد زن و 29درصد مرد) بودند. تفاوت معنی‌داری بین جنس و تعداد سقوط مشاهده شد (0/025). سقوط بیش از یکباردر سالمندان 81ساله نسبت به سایر سنین معنی‌داری بود (0/001). بین جنس و بیماری پوکی استخوان تفاوت معنی‌داری وجود داشت (0/001). همچنین نسبت شاننس بیماری پوکی استخوان در سالمندان زن، (1/663-5/804) 3/106 برابر بیشتر از سالمندان مرد بود. مردان نسبت به زنان بیشتر در معرض خطر سوءتغذیه قرار داشتند درحالی‌که این تفاوت در مورد سوءتغذیه بر عکس بود (0/001). نوع پوشش سالمندان نامناسب و محیط فیزیکی پرخطر بود. نتیجه‌گیری: باتوجه به شیوع سقوط در زنان سالمند مبتلا به پوکی استخوان و سوء تغذیه در زنان برنامه ریزی مناسب برای کنترل و پیشگیری از بیماری ها بالاخص پوکی استخوان و سوء تغذیه بایستی صورت پذیرد.

Title: رواسازی و تعیین پایایی نسخه فارسی

http://salmandj.uswr.ac.ir/article-1-602-fa.html

اهداف: هدف این تحقیق، رواسازی و تعیین پایایی نسخه ترجمه‌شده فارسی "کارآمدی اُفتادن-فرم بین‌المللی" بود. مواد و روش‌ها: جامعه آماری تحقیق، تمامی سالمندان مرد و زن شهر اراک در سال 91-1390 و نمونه آماری، دو گروه سالمندان مرد و زن(بالای 60 سال، میانگین سنّی=6/41±68/79 سال) ساکن در اراک (203 و 120 آزمودنی، به ترتیب برای تحلیل عاملی اکتشافی و تأییدی) بود که به روش در دسترس انتخاب شد. معیار تعیین حجم نمونه، به ازای هر سؤال، حداقل 5 نفر بود. داده‌ها به وسیله نسخه فارسی «مقیاس کارآمدی افتادن-فرم بین‌المللی» جمع‌آوری شد که میزان نگرانی از افتادن را در انجام 16 فعّالیّت زندگی روزانه می‌سنجد. پایایی درونی با آلفای کرونباخ 0/98 و پایایی آزمون مجدد با ضریب همبستگی پیرسون 0/70 تأیید شد. یافته‌ها: مقیاس کیسر-میر-اُلکین، 0/965 و معنی‌دار بود که نشان دهنده کفایت نمونه است. تحلیل عاملی به روش تحلیل مؤلفه اصلی و چرخش واریماکس، به استخراج یک عامل با ارزش ویژه بیش از یک (12/474) انجامید که %77/96 واریانس را پیش‌بینی کرد. بار عاملی 16پرسش‌ بین 0/637 تا 0/859 بود. پایایی درونی عالی بود (آلفای کرونباخ 0/98) و پایایی آزمون مجدد بین 1 تا 2 هفته (36نفر و %50 زن) خوب به دست آمد(ضریب همبستگی پیرسون 0/70). عملیّات آماری با SPSS-16 انجام شد. نتیجه‌گیری: براساس نتایج، نسخه فارسی «مقیاس کارآمدی افتادن- بین المللی»، دارای روایی و پایایی قابل قبول برای جامعه سالمندان ایرانی می‌باشد و می‌تواند در شرایط پژوهشی و بالینی مورد استفاده قرار گیرد.

Title: پایایی و روایی آزمون ترسیم ساعت در سالمندان

http://salmandj.uswr.ac.ir/article-1-666-fa.html

هدف: تشخیص زودهنگام اختلالات شناختی برای به‌کارگیری به‌موقع درمان‌های جدید اهمیت ویژه‌ای دارد که این مهم بدون وجود ابزارهای ارزیابی شناختی مناسب ممکن نمی شود. آزمون ترسیم ساعت یک آزمون ساده و ارزان برای ارزیابی شناختی سالمندان است که در مراکز مراقبتی اولیه سالمندان قابل استفاده می باشد. هدف تحقیق حاضر، تعیین پایایی و روایی آزمون شناختی ترسیم ساعت در سالمندان بود. روش بررسی: برای سالمندان با سواد مراجعه کننده به انجمن آلزایمر ایران (شامل بیماران و سالمندان سالم)، با روش نمونه گیری در دسترس، در مدت 5 ماه،  دومجری آزمون ترسیم ساعت با روش امتیازبندی شولمان اصلاح شده را انجام دادند. سپس آزمون کوتاه وضعیت شناختی(MMSE) برای آنها انجام شد. برای حدود یک چهارم شرکت کنندگان،  ، هفتۀ بعد نیز آزمون ترسیم ساعت انجام شد. داده‌ها با استفاده از نرم‌افزار SPSS نسخۀ ۲۰ تحلیل شد. پایایی بین ارزیابان، با محاسبۀ ICC و پایایی آزمون - بازآزمون، با محاسبۀ ضریب توافق کاپا تعیین شد. روایی هم‌زمان آزمون ترسیم ساعت و MMSE با استفاده از ضریب هم‌بستگی اسپیرمن محاسبه شد. یافته‌ها:  ۷۴ سالمند با میانگین سنی ۷۲ سال و با نسبتِ مساوی مرد و زن در این طرح شرکت کردند. مقدار ICC برای پایایی بین ارزیابان، 0/964 با 0/001>P بود. ضریب توافق کاپا بین امتیازات آزمون ترسیم ساعت در آزمون مجدد، 0/554 بود (0/001>P). ضریب هم‌بستگی اسپیرمن برای امتیاز آزمون ترسیم ساعت و MMSE، 0/782 و در سطح 0/001>P معنی‌دار بود. نتیجه‌گیری: آزمون ترسیم ساعت در سالمندان باسواد در نمونه تهرانی، از روایی و پایایی مطلوب برخوردار است و می‌توان آن را به‌عنوان یکی از ابزارهای معتبر ارزیابی شناختی سالمندان ایرانی، به‌کار گرفت.

Title: ویژگی‌های روان‌سنجی مقیاس افسردگی سالمندان در یک نمونه ایرانی

http://salmandj.uswr.ac.ir/article-1-363-fa.html

اهداف: این پژوهش با هدف بررسی ویژگی‌های روان‌سنجی مقیاس افسردگی سالمندان صورت گرفت. مواد و روش‌ها: روش این پژوهش، توصیفی است جامعه آماری این پژوهش شامل تمام سالمندان بالای 60 سال شهرکرد در کانون بازنشستگان سازمان‌ها، ارگان‌ها و مراکز سالمندان می‌باشد. 380 نفر (113 زن و 267 مرد) به روش نمونه گیری طبقه‌ای تصادفی انتخاب گردید. به منظور گردآوری داده‌ها از مقیاس افسردگی سالمندان و مصاحبه تشخیصی جامع بین‌المللی، استفاده گردید. یافته‌ها: نتایج این پژوهش، پایایی و همسانی درونی رضایت بخشی را نشان داد. ضریب آلفای مقیاس 0/75 و ضریب پایایی مقیاس به روش دو نیمه سازی، 0/77 برآورد گردید. همچنین نتایج تحلیل ROC نشان داد که بهترین نقطه برش برای این مقیاس نمره 16/5 با حساسیت 0/88 و ویژگی 0/87 می‌باشد. نتیجه‌گیری: نتایج این پژوهش نشان داد که مقیاس افسردگی سالمندان، یک ابزار روا و پایا می‌باشد و می‌توان از آن برای تشخیص افسردگی در سالمندان استفاده نمود.

Title: تبیین سلامت سالمندان: یک مدل جمعیت‌شناختی

http://salmandj.uswr.ac.ir/article-1-557-fa.html

اهداف: ارائه یک مدل رگرسیونی برای تبیین سلامت روانی و جسمی سالمندان بر اساس محل اقامت. مواد و روش‌ها: مطالعه حاضر بر پایه همبستگی در 250 نفر از جامعه سالمندان 60 سال و بالاتر زن و مرد مقیم منزل و خانه سالمندان شهر تهران انجام شد. با توجه به اینکه حداقل حجم نمونه برای مطالعات همبستگی 100 و بیشترین تعداد موردنیاز برای آزمون همخوانی مجذور کای 200 نفر توصیه شده با احتساب افت نمونه بر پایه طرح نمونه‌برداری چند مرحله ای بصورت تصادفی انتخاب شدند. داده‌ها با استفاده از پرسش‌نامه دموگرافیک و SF-36 توسط پرسشگران آموزش دیده انجام شد. داده‌ها با استفاده از نرم‌افزار SPSS نسخه 16 مورد تجزیه‌وتحلیل آماری قرار گرفت. یافته‌ها: میانگین گروه سالمندانی که دارای درآمد ناکافی هستند در هر سه نمره سلامت جسمی، روانی و کلی به گونه معناداری کمتر از گروه سالمندان با درآمد کافی است. سالمندانی که در خانه سالمندان اقامت دارند نسبت به سالمندانی که در خانه شخصی، با خانواده و به گونه کلی به غیر از خانه سالمندان زندگی می‌کنند از سلامت جسمی و سلامت کلی کمتری برخوردارند. اما میانگین سلامت روانی در هر دو گروه با یکدیگر برابر است. سالمندان متأهل نسبت به سالمندانی که مجرد، بیوه یا مطلقه هستند در هر سه مؤلفه سلامت کلی، جسمی و روانی وضعیت مناسب‌تری دارند. با افزایش میزان تحصیلات وضعیت سلامت جسمی و روانی مناسب‌تر و بیشتر است. سالمندانی که به نحوی از فرد یا افراد دیگری مراقبت می‌کنند نسبت به آن‌هایی که در این شرایط قرار ندارند از لحاظ سلامت جسمی در وضعیت مناسب‌تری قرار دارند و میانگین آن‌ها در این دو مؤلفه بزرگ‌تر از گروهی است که مراقبت از کسی را بر عهده ندارند. این مطلب در مورد سالمندانی که تحت مراقبت قرار دارند معکوس است. بدین‌ترتیب که سالمندانی که تحت مراقبت فرد یا افرادی قرار دارند از لحاظ سلامت جسمی و نمره کل سلامتی از افرادی که تحت مراقبت قرار ندارند به گونه معناداری کمتر است. نتیجه‌گیری: متغیرهای دموگرافیک هم ارز دیگر متغیرهای جسمی و روانی می‌توانند وضعیت سلامت سالمندان را پیش‌بینی کنند. برای دستیابی به سالمندی موفق در آینده سیاست‌گذاران می‌توانند روی متغیرهای پیش‌بینی‌کننده سلامت سالمندان متمرکز شوند.

Title: معنویت در تجربیات سالمندان دارای فرزند معلول ذهنی-یک پژوهش با رویکرد پدیدارشناسی

http://salmandj.uswr.ac.ir/article-1-556-fa.html

اهداف: با توجه به افزایش طول عمر معلولین ذهنی و نیز والدین آنان پیش‌بینی می‌شود در طی سی سال آینده یک رشد قابل توجه در تعداد سالمندانی وجود داشته باشد که از فرزند معلول خود نگهداری می‌کنند. این درحالی است که هنوز مطالعه کافی به منظور بررسی تجارب والدین سالمند دارای فرزند معلول ذهنی انجام نشده است. مقاله حاضر بخشی از یک مطالعه با رویکرد پدیدارشناسی توصیفی است که به بررسی تجربیات سالمندان دارای فرزند معلول ذهنی با تمرکز بر موضوع معنویت پرداخته است. مواد و روش‌ها: مطالعه کیفی حاضر با استفاده از رویکرد پدیدار شناسی از طریق انجام ده مصاحبه بدون ساختار عمیق با والدین سالمند دارای فرزند معلول ذهنی انجام گرفته است. پنج نفر از مصاحبه شوندگان زن و پنج نفر مرد بودند و داده‌ها با روش هفت مرحله‌ای کلایزی تحلیل شده است. یافته‌ها: چهار مضمون اصلی از داده‌ها استخراج گردید که یکی از آنها را مضمون اصلی معنویت تشکیل می داد. سه مضمون اصلی دیگر "تلخ‌کامی" ، "دلبستگی"، "رضایت از حمایت" بود. این والدین اگر چه دارای مشکلاتی در رابطه با مراقبت از فرزندان معلول ذهنی می‌باشند و آلام زیادی را تجربه کرده اند ولی از طرف دیگر وجود فرزند معلول را عامل این مشکلات نمی‌دانند. آن‌ها وجود مددجو را خواست خدا دانسته و حتی با شرایط فعلی شکرگزار بودند. مقاله حاضر به توصیف مضمون "معنویت" می پردازد. نتیجه‌گیری: معنویت در تجربیات سالمندان شرکت‌کننده در این مطالعه کاملا بارز است. به‌طور عمده آنان حضور فرزند معلول ذهنی را خواست خداوند و حتی نعمتی از طرف او می‌دانند. این دیدگاه‌ها می‌توانند به مشکلات معنای متفاوتی ببخشد. به‌طوری که والدین این مشکلات را به عنوان امتحان الهی بدانند و دراین میان رنج‌ها قابل تحمل‌تر و حتی دارای ارزش می‌شود. لذا لازم است در برنامه‌های حمایتی این گروه از سالمندان به جنبه‌های معنوی تجربیات ایشان توجه شود و با توجه به اینکه زندگی این سالمندان پدیده‌ای فرایندی می‌باشد توصیه می‌گردد به روش نظری پایه این فرایند مورد بررسی عمیق قرار گیرد.
